# When microsurgery is not an option: staged delayed reverse sural artery flap with ischaemic preconditioning for limb salvage in an elderly patient with chronic post-traumatic osteomyelitis—a case report

**DOI:** 10.3389/fsurg.2026.1894777

**Published:** 2026-09-03

**Authors:** Zhun Shen Tan, Adzim Poh Yuen Wen, Farzana Ansarutheen, Mohd Yazid Bajuri

**Affiliations:** 1 Faculty of Medicine, Universiti Kebangsaan Malaysia, Kuala Lumpur, Malaysia; 2 Plastic Surgery Unit, Department of Surgery, Faculty of Medicine, Universiti Kebangsaan Malaysia, Kuala Lumpur, Malaysia; 3 Department of Orthopaedics and Traumatology, Faculty of Medicine, Universiti Kebangsaan Malaysia, Kuala Lumpur, Malaysia

**Keywords:** ankle reconstruction, case report, delay phenomenon, exposed calcaneus, ischemic preconditioning, lower limb salvage, osteomyelitis, reverse sural artery flap

## Abstract

Lower extremity reconstruction in elderly patients with post-traumatic osteomyelitis presents a formidable challenge. We report a 70-year-old female with hypertension, dyslipidaemia, and bilateral knee osteoarthritis who sustained a Gustilo-Anderson type IIIB open right ankle fracture with exposed calcaneus following a fall. The course was complicated by chronic osteomyelitis, managed with partial talectomy and total calcanectomy, antibiotic-impregnated cement spacer placement, cement exchange and prolonged culture-directed intravenous antibiotics. Following infection eradication, definitive hindfoot fusion using a structural femoral head allograft fixed with a retrograde tibiotalocalcaneal arthrodesis nail and compression screws was performed. However, a persistent soft-tissue defect with exposed bone precluded primary closure. Given her age, comorbidities and compromised local vascularity, free tissue transfer was deemed high-risk. Therefore, a 2-stage delayed reverse sural artery flap (RSAF) was selected. The first stage involved flap elevation with ischaemic preconditioning over 2 weeks via stepwise incremental incisional delay, followed by definitive transposition and inset. The donor site was managed with an ovine tendon collagen (OTC) matrix and split-thickness skin grafts (STSGs), supported by intermittent negative-pressure wound therapy (NPWT). At 6-months of follow-up, the flap remained viable and had fully survived, and the patient achieved partial weight-bearing ambulation. This case illustrates the decision-making framework for complex lower limb salvage, highlighting a staged reconstructive strategy integrating the delay phenomenon and adjunctive wound technologies to achieve durable limb salvage when microsurgical reconstruction is contraindicated.

## 1 Introduction

Soft tissue reconstruction of the distal lower extremity remains one of the most demanding challenges in reconstructive surgery, particularly in elderly patients with comorbidities (1, 2). The ankle and hindfoot are especially vulnerable due to limited local soft tissue, tenuous vascularity and frequent exposure of bone and tendons following trauma or repeated debridement. These challenges are compounded in post-traumatic osteomyelitis, where definitive soft tissue coverage can only be safely pursued after complete infection eradication and premature flap coverage associated with high rates of flap failure, wound breakdown and recurrent infections (3). Free tissue transfer is the preferred method of coverage for such defects, typically with anterolateral thigh (ALT) or latissimus dorsi (LD) flaps (4, 5). However, compromised recipient vessels, reduced cardiopulmonary reserve, prolonged operative time and significant medical comorbidities may render microsurgical reconstruction unsuitable in elderly patients (6, 7).

In such circumstances, regional pedicled flaps offer a reliable alternative. The reverse sural artery flap (RSAF) is a distally based neurocutaneous flap that provides dependable coverage for defects of the distal third of the leg, ankle and heel without sacrificing major axial arteries to the foot, perfused via retrograde flow through peroneal artery perforators along the vascular axis of the sural nerve and lesser saphenous vein (8, 9). The RSAF maintains reliable vascularity via the sural angiosome (10), and staged elevation with incorporation of the delay phenomenon further improves flap reliability in high-risk patients with compromised microcirculation (11). The delay phenomenon, or ischaemic preconditioning, exploits controlled sublethal ischaemia to recruit choke vessels between adjacent angiosomes and to upregulate angiogenic mediators, progressively converting a random-pattern territory into a more robustly perfused axial one (11).

We present a case of complex post-traumatic ankle reconstruction using a 2-stage delayed RSAF in an elderly patient with chronic osteomyelitis and exposed calcaneus following hindfoot fusion, focusing on the decision-making framework, technical considerations of ischaemic preconditioning (IPC), and the role of adjunctive therapies in achieving successful limb salvage.

## 2 Case report

### 2.1 Patient presentation and initial management

**Figure 1:**
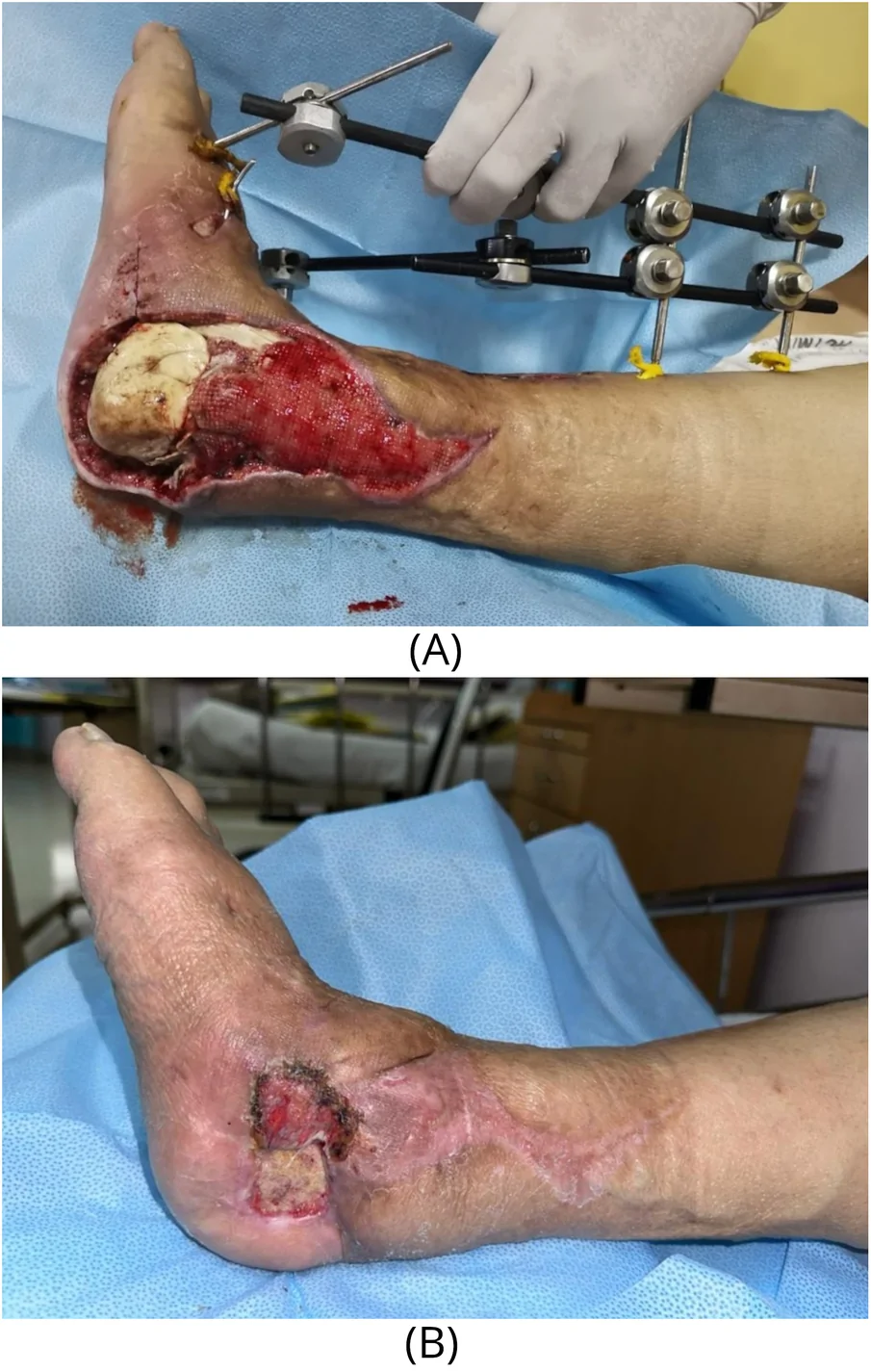
**(A)** initial presentation of the gustilo-anderson type IIIB open right ankle fracture with cross-ankle-joint external fixation *in situ*. **(B)** Right lower limb at referral to plastic surgery, showing a chronic granulomatous soft-tissue wound with an underlying chronic osteomyelitic wound at the distal aspect.

**Figure 2:**
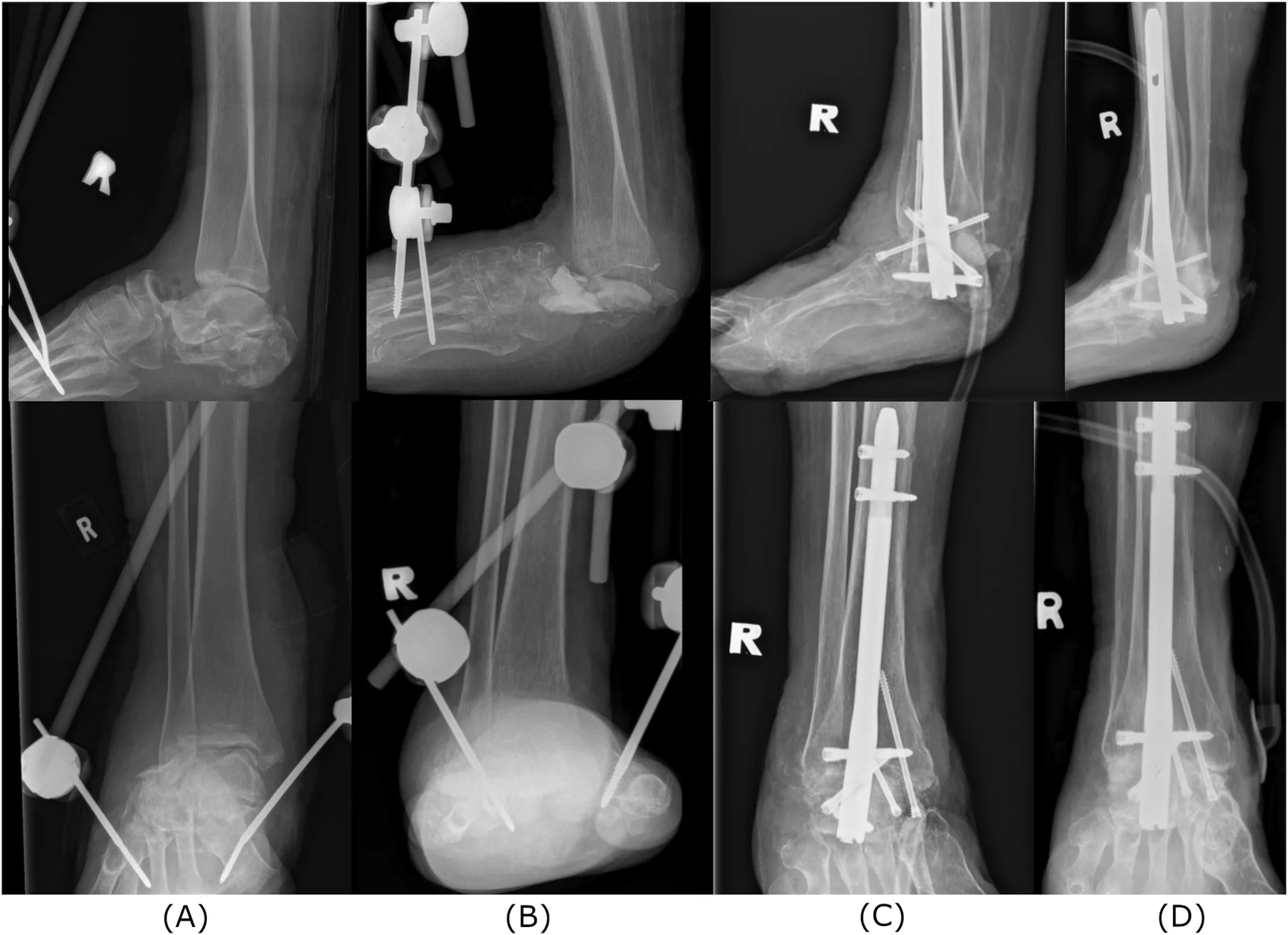
Staged radiographic documentation. **(A)** Initial injury with cross-ankle-joint external fixation. **(B)** Antibiotic-impregnated cement spacer *in situ* following partial talectomy and total calcanectomy. **(C)** Definitive hindfoot arthrodesis with structural femoral head allograft and a retrograde tibiotalocalcaneal arthrodesis nail. **(D)** Follow-up radiograph at 6 months.

A timeline summarising the key clinical events is provided in Table 1. A 70-year-old Chinese female with a background of hypertension, dyslipidaemia, and bilateral knee osteoarthritis with a history of left total knee replacement sustained a fall resulting in multiple lower limb injuries. Initial assessment revealed an open right ankle fracture classified as Gustilo-Anderson type IIIB with extensive soft tissue loss and exposed calcaneus (Figure 1A), along with an open left periprosthetic tibial fracture and closed fractures of the left calcaneus and talus. She was haemodynamically stable and underwent emergency stabilisation with cross-ankle-joint external fixation and multiple wound debridements of the right ankle (Figure 2A).

**Table 1:** Timeline of clinical course and reconstructive management.

Time from presentation	Clinical event
0 months	Fall resulting in Gustilo-Anderson type IIIB open right ankle fracture with exposed calcaneus, open left periprosthetic tibial fracture and closed left calcaneal and talar fractures. Emergency cross-ankle-joint external fixation and initial wound debridements were performed
0–3 months	Serial debridements and VAC therapy were undertaken, complicated by the development of chronic osteomyelitis with persistently elevated inflammatory markers and polymicrobial wound cultures
3 months	Admission for intensive wound care following identification of a 4 × 2 cm area of exposed calcaneum with tendon exposure
4–5 months	Partial talectomy and total calcanectomy, with antibiotic-impregnated cement spacer placement, followed by 6 weeks of culture-directed intravenous antibiotics
9 months	Repeat debridement and antibiotic cement exchange. Progressive normalisation of inflammatory markers was observed
11–14 months	Stable granulation tissue achieved. Persistent cement exposure precluded early definitive skeletal reconstruction
15 months	Right hindfoot fusion with a structural femoral head allograft, and a retrograde tibiotalocalcaneal arthrodesis nail, performed following cement removal
15–16 months	Healthy granulation with persistent soft-tissue tenuousness. Referral to plastic surgery for definitive coverage
17 months	1st Stage: Reverse sural artery flap elevation with preservation of lesser saphenous vein and sural nerve. The flap was returned to its donor bed with serial stepwise incisional extension over 2 weeks for ischaemic preconditioning
17.5 months	2nd Stage: Definitive flap transposition and inset performed. Residual recipient defects were resurfaced with a full-thickness skin graft (FTSG) harvested from excised native flap tissue. The donor site was managed with ovine tendon collagen (OTC) matrix and split-thickness skin grafts (STSGs)
19–21 months	Complete flap and skin graft take. Non-weight-bearing rehabilitation commenced
21–23 months	Viable, well-healed flap with <0.5 cm residual superficial wound. No recurrent infection, underlying implants stable. The patient progressed to partial weight-bearing ambulation
Outcome	Durable limb salvage with functional ambulation

Despite aggressive early management, the patient developed chronic osteomyelitis of the right hindfoot. Serial inflammatory markers remained persistently elevated, and wound cultures confirmed polymicrobial infection. Serial assessments documented exposed bone and tendons over the right hindfoot, unhealthy slough, and recurrent infection despite repeated vacuum-assisted closure (VAC) therapy, antimicrobial dressings and systemic antibiotics. By approximately 3 months from presentation, a 4 × 2 cm area of exposed calcaneum with tendon exposure was identified, prompting admission for intensive wound care. Persistent seropurulent discharge and elevated inflammatory markers confirmed ongoing osteomyelitis, necessitating operative resection of the affected hindfoot bone with placement of an antibiotic-impregnated cement spacer (Figure 2B), followed by 6 weeks of culture-directed intravenous antibiotic therapy. Repeat surgical debridement and antibiotic cement exchange were subsequently performed. Intraoperative findings confirmed osteomyelitis involving both the talus and the calcaneus. The osteomyelitic talus was debrided back to punctate bleeding (“paprika sign”) viable bone and a partial talectomy performed, preserving viable residual talar bone, whereas the calcaneus was completely excised (total calcanectomy). Radical excision of the calcaneus, rather than partial resection, was appropriate given that more than 50% of its body was involved. The talus was only partially resected to preserve reconstructable talar bone stock and avoid enlarging the segmental defect. Serial infection-control debridements preceded definitive reconstruction, which was deferred until cultures and inflammatory markers confirmed control so that a structural allograft was not introduced into an actively infected bed. Serial monitoring thereafter demonstrated progressive infection control, with normalisation of inflammatory markers and resolution of purulent discharge. However, cement exposure persisted, precluding early definitive skeletal reconstruction.

### 2.2 Hindfoot reconstruction

At approximately 15 months from presentation, the wound demonstrated stable granulation tissue without overt signs of infection, though bone cement remained exposed. Following multidisciplinary optimisation, the patient underwent right hindfoot fusion after cement removal (Figure 2C). The osseous defect created by the total calcanectomy and partial talectomy was reconstructed with a single structural femoral head allograft (LifeNet Health) and fixed with a retrograde tibiotalocalcaneal arthrodesis nail (11 mm × 200 mm) with locking screws, supplemented by compression screws. The nail passed retrograde through the femoral head allograft, which occupied the calcaneal position and the residual talus into the distal tibia. The allograft restored hindfoot height and provided a stable osseous platform beneath the heel pad, maintaining heel-pad viability and enabling plantigrade weight-bearing through the fused foot. She was discharged postoperatively with a portable VAC dressing. Subsequent wound inspection demonstrated healthy granulation tissue with no bone exposure or purulent discharge. However, soft tissue coverage remained tenuous and the complex reconstructive history necessitated referral to the plastic surgery team for definitive soft tissue reconstruction (Figure 1B).

### 2.3 Surgical planning and reconstructive technique

Given the patient's advanced age, multiple comorbidities, bilateral lower limb injuries, history of prolonged infection and compromised local vascularity, free tissue transfer was deemed excessively high-risk. The risks of anastomotic thrombosis, prolonged anaesthesia, and donor site morbidity were considered unacceptable. Intraoperative assessment confirmed that the peroneal artery perforators had been avulsed at the time of the original injury, leaving the distal soft tissues perfused only via peripheral cutaneous (random-pattern) blood flow. This compromised perforator anatomy provided the principal rationale for adopting a staged delayed approach with IPC rather than immediate transposition. After thorough multidisciplinary discussion, a 2-stage delayed reverse sural artery flap (RSAF) was selected to optimise perfusion and minimise the risk of flap failure.

**Figure 3:**
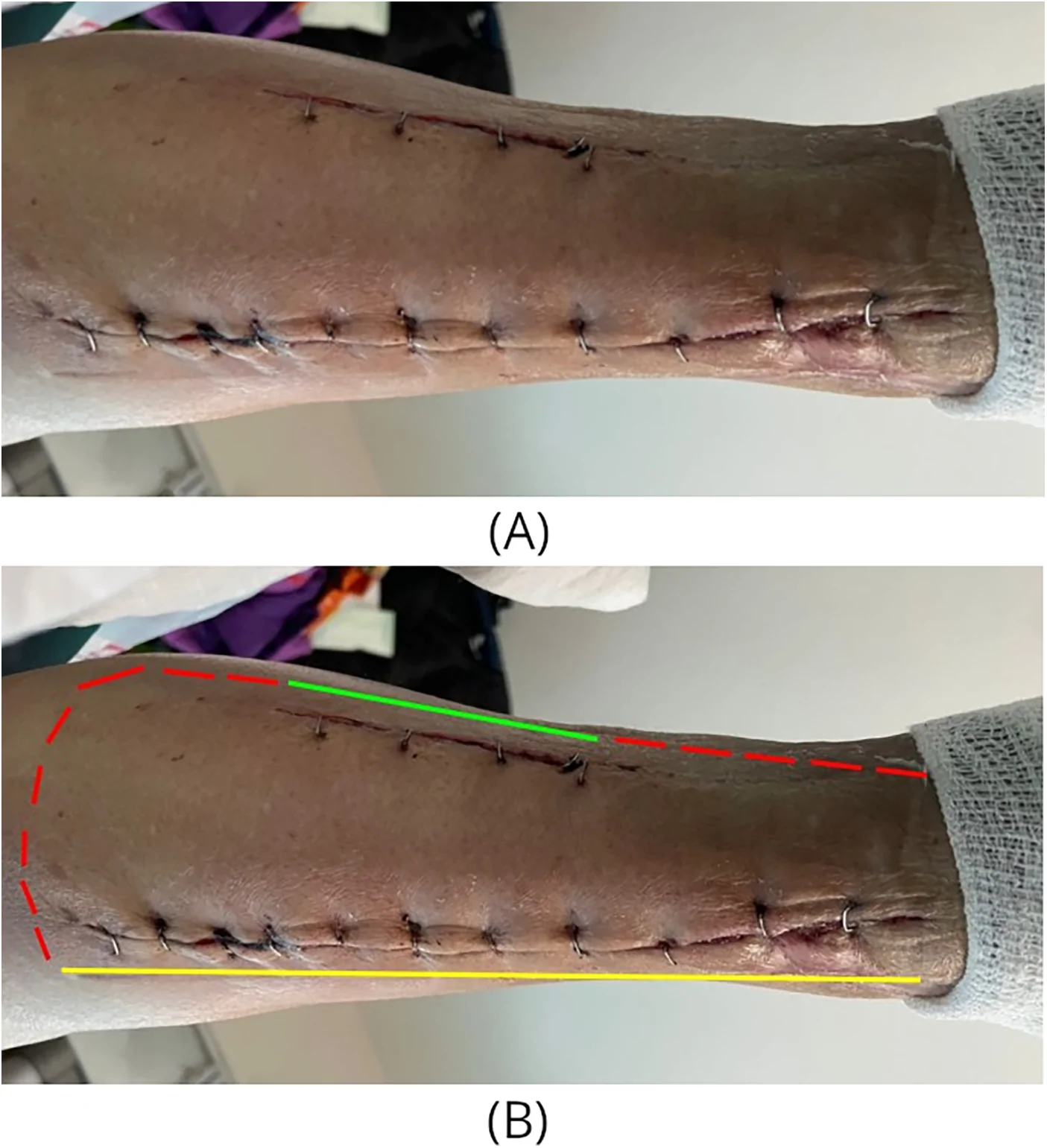
1st stage flap design and incremental delay of the reverse sural artery flap. **(A)** Plain photograph of the flap design. **(B)** Annotated view: the medial limb of the incision (marked by a solid yellow line) and the lateral limb (marked by a solid green line), together defining 2 halves of a subcutaneous flap in a 1:1 ratio. The stepwise cephalad and caudal extensions performed every 3 to 4 days (indicated by the multiple red lines), continued until the cephalad incision completes a semicircle, with the lateral limb mirroring the length of the medial limb to progressively isolate the flap and recruit choke vessels along the sural axis.

**Figure 4:**
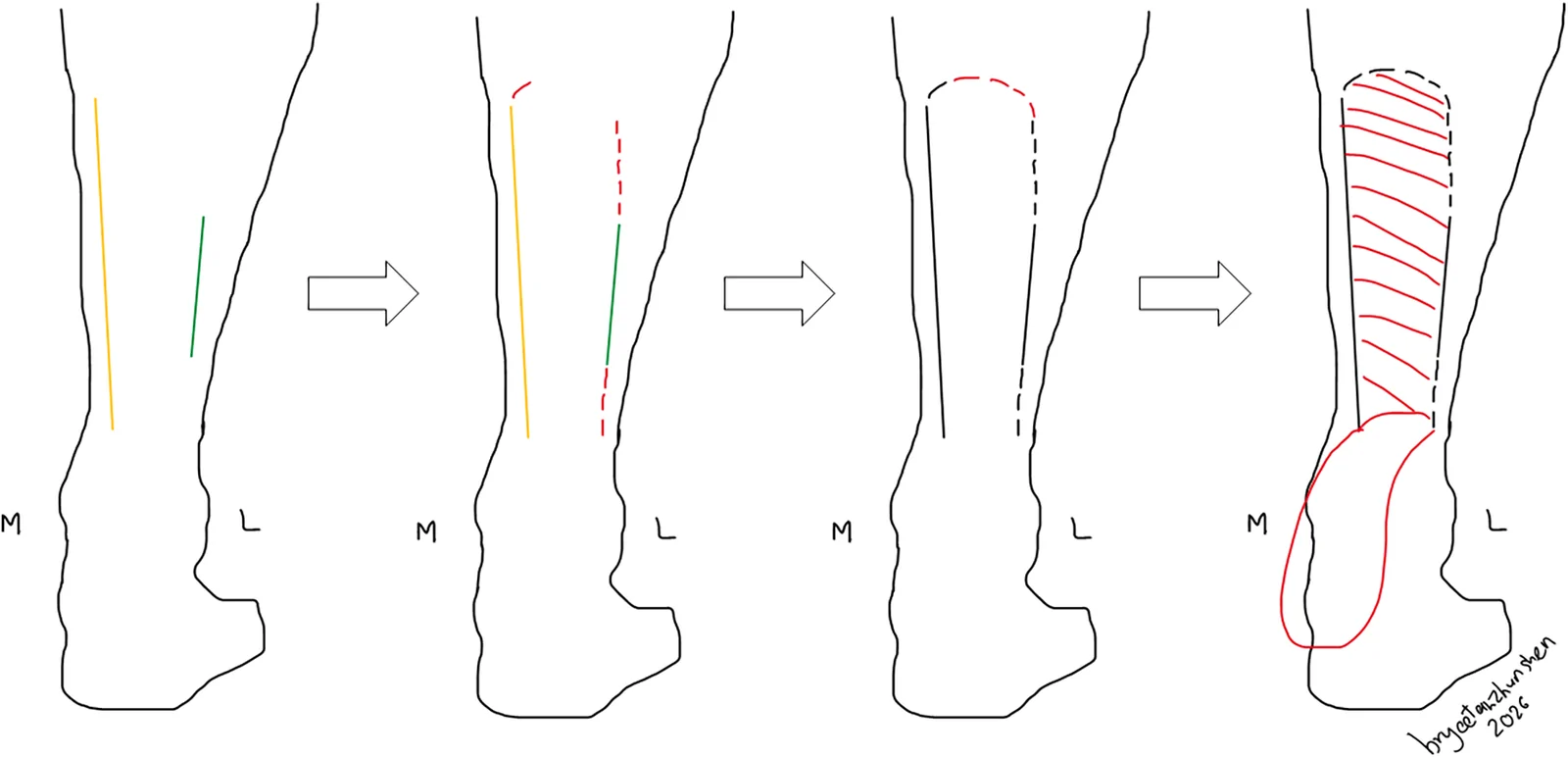
Schematic illustration of the stepwise incisional delay of the reverse sural artery flap over the 2-week preconditioning period, showing the incision advancing and the progressive recruitment of choke vessels along the sural axis.

The 1st stage involved flap design and elevation under general anaesthesia, based on retrograde flow from the peroneal artery perforators, with preservation of the lesser saphenous vein and sural nerve within the flap. Following formal anaesthetic assessment, the patient was deemed fit for general anaesthesia at very low risk. General anaesthesia was selected in preference to regional or local techniques because each stage involved concurrent extensive debridement in addition to flap surgery, for which regional or local anaesthesia would not have provided adequate operative conditions. This also reflected surgeon and patient preference. The flap was designed as 2 halves of a subcutaneous flap in a 1:1 ratio, demarcated by a medial limb and a lateral limb of incision, on the assumption that the vascularity of the medial limb was robust while that of the lateral limb was comparatively poor. Rather than transferring the flap immediately, the incised flap was stapled back across the full length of the incision to its donor bed and intentionally delayed. Over the following 2 weeks, the incision was serially extended in a stepwise fashion both cephalad and caudally every 3 to 4 days, continued until the cephalad incision completed a semicircle and the lateral limb mirrored the length of the medial limb. This incremental incisional delay induced ischaemic preconditioning and promoted compensatory angiogenesis along the sural vascular axis, progressively augmenting perfusion across the less reliable lateral territory before definitive transfer (Figures 3A,B**)**. The stepwise incisional delay over the two-week preconditioning period is illustrated schematically in Figure 4.

**Figure 5:**
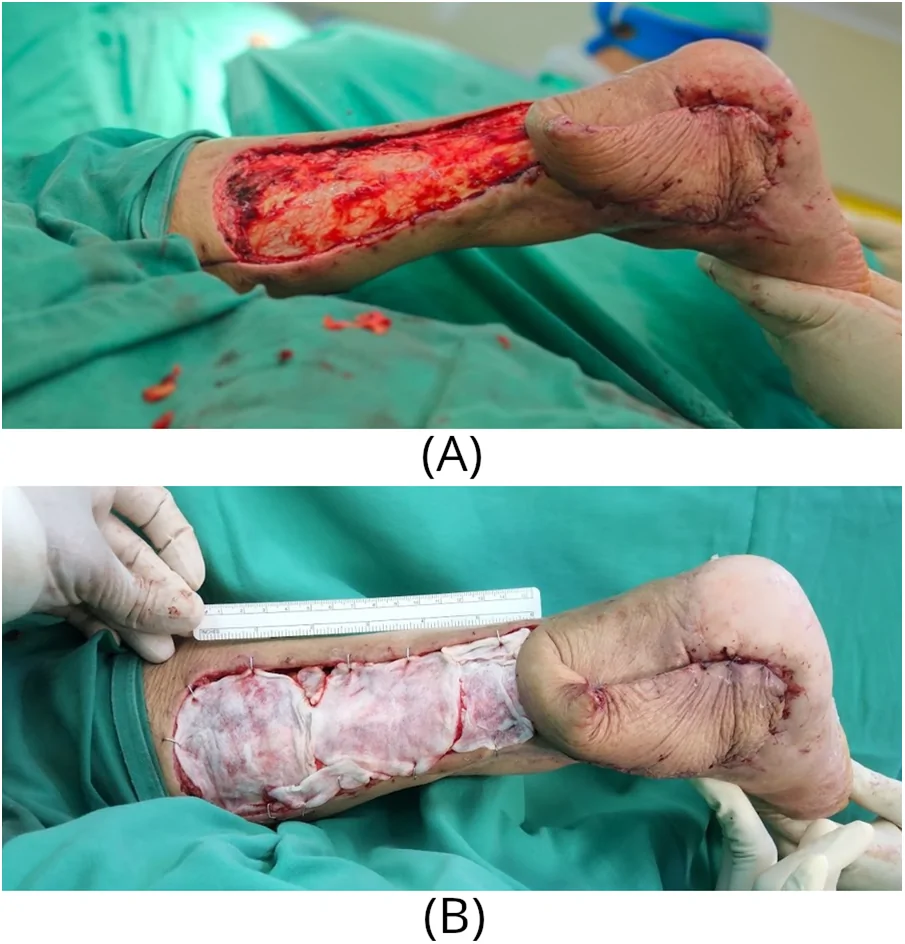
2nd stage reconstruction. **(A)** Definitive transposition and inset of the elevated flap over the recipient hindfoot defect. **(B)** Posterior calf donor site managed with an OTC matrix.

The 2nd stage proceeded after confirming robust distal capillary refill and bleeding from the flap margins consistent with adequate perfusion. The flap was elevated, transposed, and inset to provide stable, tension-free coverage over the exposed hindfoot (Figure 5A). A small residual defect at the recipient site was resurfaced using a full-thickness skin graft (FTSG) harvested from the excised native sural flap tissue, maximising local tissue utilisation. The posterior calf donor site was managed with an ovine tendon collagen (OTC) matrix to provide a biologically favourable dermal scaffold **(**Figure 5B**)**, followed by split-thickness skin grafting (STSG) from the ipsilateral thigh. Intermittent negative-pressure wound therapy (NPWT) was applied to both recipient and donor sites to optimise graft take and wound bed stabilisation throughout the postoperative period.

### 2.4 Postoperative course and outcomes

The immediate postoperative course was uneventful. At 2 weeks, both the flap and skin grafts demonstrated complete take with no evidence of venous congestion, arterial insufficiency, infection, or wound dehiscence. The patient was discharged with strict non-weight-bearing instructions.

**Figure 6:**
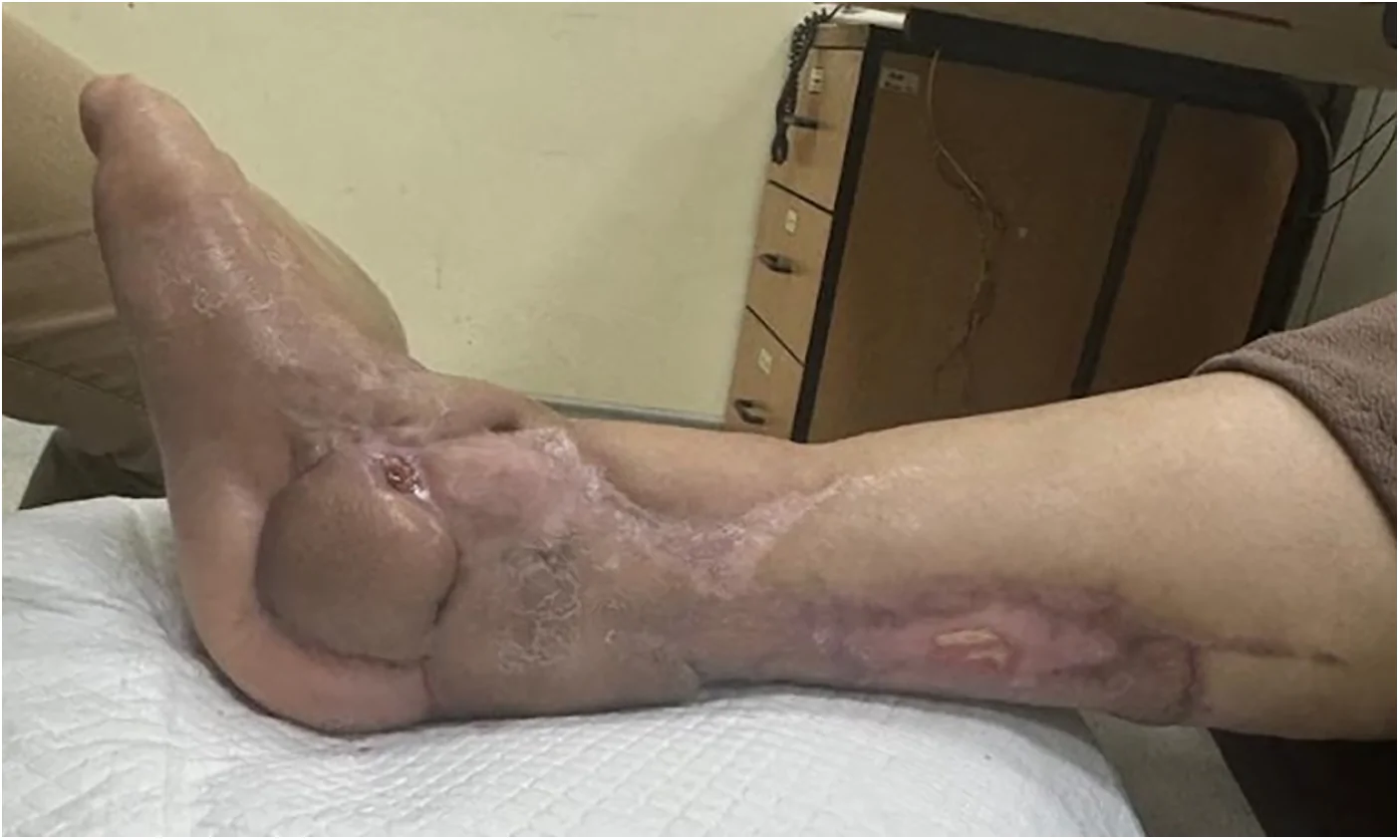
Healed reconstruction at 6 months, showing a viable, well-settled flap with resolved wound and no recurrent infection.

At 6 months following flap reconstruction, the flap remained viable and well-healed, with only a small residual superficial wound measuring less than 0.5 cm at the flap margin which was managed conservatively with dressings and intermittent VAC therapy as required. There was no evidence of distal tip necrosis, epidermolysis, or recurrent infection. Inflammatory markers remained within the normal range and the fixation construct remained stable on plain radiographs (Figure 2D). As the reconstruction comprised a hindfoot arthrodesis, the ankle was surgically fused by design; ankle range of motion is therefore not applicable, and the patient ambulated with a plantigrade, stable foot. The patient was ambulating with partial weight-bearing with tolerable pain and demonstrated satisfactory functional progress (Figure 6).

## 3 Discussion

### 3.1 Infection control as a reconstructive prerequisite

Reconstruction of distal lower limb defects in elderly patients with post-traumatic osteomyelitis remains a formidable clinical challenge. In this case, exposed calcaneus, compromised local tissue quality and prior infection markedly constrained the reconstructive options and increased the risk of flap failure (1, 4). The reconstructive strategy was guided by the fundamental principle that durable soft tissue coverage can only be reliably achieved after definitive infection eradication. Chronic osteomyelitis necessitates radical excision of all necrotic bone and sequestrum. Without complete bacterial clearance, any subsequent flap reconstruction is liable to fail due to persistent colonisation of bone or soft tissue (3).

The osteomyelitis in this case was a direct sequelae of the high-energy, contaminated type IIIB open injury and was already established when the patient was referred to our unit approximately 1 year after the index trauma. It was therefore not a consequence of delayed coverage. Throughout the pre-coverage interval the wound was protected with NPWT, and the definitive 2-stage delayed flap added only approximately 3 weeks to the overall course. Premature coverage of a colonised bed carries a high risk of flap failure and recurrent infection (3). Moreover, the peroneal perforators had been avulsed by the index trauma, so a durable distally based local flap was not available until the staged delay re-established axial perfusion.

Our patient required sequential surgical intervention spanning nearly a year (including total calcanectomy and partial talectomy, repeated debridements, antibiotic cement spacer placement, cement exchange and ultimately allograft hindfoot fusion) before the wound environment was deemed suitable for flap coverage. This stepwise, infection-first approach, while prolonged, was essential to ensure a viable wound bed and prevent the cascade of flap failure and recurrent infection that would otherwise have resulted from premature reconstruction.

### 3.2 reconstructive decision-making: free flap vs. pedicled option

Although free tissue transfer is the preferred option for distal lower-limb defects (2, 4, 5), its success depends on adequate recipient vessel quality, prolonged operative tolerance and reliable microsurgical conditions. In elderly patients with multiple comorbidities and compromised vascularity, free flaps carry substantially increased risks of anastomotic thrombosis, prolonged anaesthesia and donor site morbidity. It is this comorbidity burden, rather than chronological age itself, that escalates the risk of microsurgical reconstruction (7). This was a decisive consideration in the present case.

The RSAF was selected as the optimal reconstructive option based on several compelling factors. The flap provides reliable vascularity via the sural angiosome without sacrificing any major axial artery to the foot, thereby preserving future reconstructive options which is a critical consideration in a patient with bilateral lower limb injuries and the attendant risk of contralateral compromise (10). The RSAF offers a greater arc of rotation than other regional pedicled alternatives such as the peroneal artery perforator flap, permitting tension-free coverage of distal defects including the exposed calcaneus (12). Furthermore, institutional constraints including high patient volume and limited immediate microsurgical resources necessitated shorter operative times, making the RSAF a practical and appropriate choice (4).

It should be emphasised that advanced age alone does not preclude major reconstruction. Current evidence indicates that age alone bears no significant correlation with free or pedicled flap failure rates when patients are carefully selected and appropriately optimised (7). The decision in this case was therefore driven not by age exclusion, but by the constellation of local and systemic risk factors that rendered microsurgical reconstruction disproportionately hazardous for this specific patient.

### 3.3 The delay phenomenon and 2-stage approach

The decision to employ a 2-stage rather than single-stage RSAF was justified by the patient's advanced age, underlying comorbidities, bilateral injuries, and the hostile nature of the wound bed following prolonged infection and repeat surgery. While a single-stage RSAF may offer the advantage of a shorter hospital stay and reduced operative burden, it carries a well-documented risk of partial tip necrosis, epidermolysis, and the need for secondary procedures (13). These complications are particularly pronounced in patients with compromised microcirculation, chronic infection, and impaired tissue quality (11). All of these factors were present in the current case.

To further improve flap survival, IPC, also known as the delay phenomenon, was incorporated into the reconstructive strategy (11). This was achieved by initially elevating the flap, returning it to its donor bed, and performing serial stepwise incisional delay of its base over the subsequent 2 weeks. At a molecular level, sublethal ischaemia upregulates hypoxia-inducible factor-1*α* and vascular endothelial growth factor, drives choke-vessel dilatation and neovascularisation between angiosomes, and confers anti-apoptotic, anti-oxidative and anti-inflammatory protection to the preconditioned tissue (11). Clinically, this augments perfusion of the distal flap territory most at risk of necrosis, which is of particular value in an elderly patient with reduced physiological reserve, a previously infected bed, and traumatically avulsed peroneal perforators. This process promotes compensatory angiogenesis and vasculogenesis along the axial vessel of the sural artery, creating a robust axial blood supply in territory that was previously dependent on random perfusion (11). IPC has been demonstrated to significantly increase flap survival, particularly in ischaemic or traumatised tissue beds such as those compromised by prior infection or systemic disease (11).

The cautious, staged approach employed in this case reflects an appropriate alignment between reconstructive ambition and patient-specific risk. By deferring definitive flap inset until robust distal perfusion was confirmed, we minimised the likelihood of catastrophic flap loss in a setting where reoperation would carry substantial morbidity.

### 3.4 Adjunctive therapies

Adjunctive therapies played an important supportive role at multiple stages of the reconstructive pathway. NPWT was utilised intermittently throughout the perioperative period, promoting granulation tissue formation, reducing interstitial oedema, lowering bacterial bioburden, and improving local perfusion (14, 15). Its application to both the recipient and donor sites facilitated graft take in an otherwise challenging wound environment.

For donor site management, an OTC matrix was applied prior to STSG. This biologically active scaffold promotes cellular infiltration and dermal regeneration, and has demonstrated efficacy in compromised wound beds including those with prior infection, radiation injury, or poor vascularity (16). Because the donor-site wound bed lay directly over exposed gastrocnemius muscle, application of the OTC matrix, a dermal regenerative template, provided a vascularised bridging layer over the muscle that promoted cellular infiltration and neodermal formation, creating a receptive bed for successful STSG take where straightforward grafting would otherwise have been unreliable.

The decision to harvest the FTSG for residual recipient site coverage from excised native sural flap tissue was both practical and resourceful, avoiding additional donor site morbidity while maximising the use of available tissue.

### 3.5 Limitations and future considerations

The RSAF is not without recognised limitations. Venous congestion remains a well-documented complication, particularly in flaps exceeding 10–12 cm in length or in patients with compromised peripheral venous return (10, 13). In this case, venous complications were mitigated through careful flap design, deliberate preservation of the lesser saphenous vein within the pedicle, and meticulous avoidance of pedicle torsion during transposition. Sensory disturbance over the lateral foot and donor site morbidity remain documented in the literature, though neither was observed in our patient at the time of reporting.

The use of a structural femoral head allograft to reconstruct a previously osteomyelitic hindfoot is a recognised high-risk strategy, given the potential for non-incorporation and infection recurrence. Furthermore, the six-month follow-up reported here, although free of clinical or biochemical recurrence, is relatively short for chronic osteomyelitis, which is well recognised to recur months to years after apparent resolution; definitive eradication therefore cannot yet be confirmed. Our current assessment rests on normalised inflammatory markers, absence of clinical recurrence, and stable soft-tissue coverage, and longer-term clinical, biochemical and radiographic surveillance is ongoing and will be required to confirm durable infection control and allograft incorporation.

A dedicated true lateral hindfoot projection was not available in the imaging archive, and partial resorption of the allograft segment posterior to the fixation limits radiographic assessment of the hindfoot. This is acknowledged as a limitation of the radiographic documentation.

While short-term outcomes in this case were favourable, secondary procedures such as contour refinement or flap debulking may be required in the future to optimise footwear fitting and long-term functional outcomes. Extended follow-up will be necessary to confirm the durability of both the soft-tissue reconstruction and the skeletal construct. The absence of formal patient-reported outcome measures at this stage represents a limitation of this report, and future cases of similar complexity should incorporate validated functional and quality-of-life assessments.

## 4 Conclusion

This case demonstrates that a 2-stage delayed RSAF incorporating IPC remains a valuable, reliable and technically feasible reconstructive option for complex ankle and hindfoot defects in elderly patients with post-traumatic osteomyelitis when free tissue transfer is contraindicated. Successful outcomes require careful patient selection, a systematic infection-first approach, strategic application of the delay phenomenon to enhance flap perfusion and judicious integration of adjunctive wound care technologies. Patient-centred decision-making guided by individual risk stratification rather than age alone is paramount in achieving durable limb salvage in this challenging population.

## Data Availability

The original contributions presented in the study are included in the article/Supplementary Material, further inquiries can be directed to the corresponding authors.
